# Esophageal resonance Raman spectroscopy tracks mixed venous oxygenation in experimental shock and hypoxemia

**DOI:** 10.1186/s40635-026-00989-z

**Published:** 2026-10-15

**Authors:** Tysen DeWaard, Bonne L. Keijer, Franklin Ducatez, Cassidy Ferron, Jason Zhang, Yifeng Peng, Padraic Romfh, John N. Kheir

**Affiliations:** 1 Department of Cardiology, Division of Cardiovascular Critical Care, Boston Children’s Hospital, 300 Longwood Avenue, Boston, 02115 MA USA; 2 Department of Pediatrics, Harvard Medical School, Boston, 02115 MA USA; 3 Pendar Medical, LLC, Cambridge, 02138 MA USA

**Keywords:** Resonance Raman spectroscopy, Hemorrhagic shock, Hypoxia, Mixed venous oxygen saturation

## Abstract

**Introduction:**

Mixed venous oxygen saturation (SvO_2_) reflects the balance between systemic oxygen delivery and consumption and is widely used as an indicator of tissue oxygenation in critically ill patients. However, its measurement requires invasive venous access. We evaluated whether a customized probe applied to the esophageal mucosa could provide a continuous measurement of regional capillary hemoglobin oxygen saturation (S_Es_O_2_) that tracks changes in mixed venous oxygen saturation for early detection of experimental shock with minimal invasiveness.

**Methods:**

A miniaturized 405 nm resonance Raman spectroscopy (RRS) probe was developed to quantify esophageal regional capillary hemoglobin saturation (S_Es_O_2_) based on spectral decomposition of oxy- and deoxy-hemoglobin Raman peaks. Two rat models were investigated: progressive hemorrhage (n = 12) and graded hypoxia (n = 9). At each stage, SvO_2_, S_Es_O_2_, arterial oxygen saturation (SaO_2_), and serum lactate were measured. Tracking ability and diagnostic accuracy compared to SvO_2_ and transabdominal near-infrared spectroscopy (NIRS) were evaluated using linear mixed-effects models (animal random intercepts), (cluster-bootstrapped) receiver operating characteristic curve (ROC) analyses, and Pearson correlation.

**Results:**

In both models, baseline S_Es_O_2_ was ~20% higher than SvO_2_, consistent with a regional capillary measurement distinct from mixed venous blood. In hemorrhage, LME showed S_Es_O_2_ was positively associated with SvO_2_ (β = 0.53, 95% CI 0.43–0.62; p < 0.0001) and inversely associated with lactate (β = −0.15, 95% CI −0.18 to −0.12; p < 0.0001). In ROC analysis, S_Es_O_2_ discriminated SvO_2_ < 40% with AUC 0.98 (95% CI 0.95–1.00). For transabdominal NIRS, the AUC was 0.82 (95% CI 0.70–0.95). During graded hypoxia, S_Es_O_2_ was also positively associated with SvO_2_ (β = 0.51, 95% CI 0.43–0.60; p < 0.0001) and inversely associated with lactate (β = −0.11, 95% CI −0.13 to −0.09; p < 0.0001). Discrimination of S_Es_O_2_ for SvO_2_ < 40% was AUC 0.92 (95% CI 0.82–1.00). Transabdominal NIRS demonstrated discrimination with high uncertainty (AUC 0.56, 95% CI 0.23–1.00).

**Conclusions:**

Esophageal RRS enables molecularly specific measurement of regional capillary hemoglobin oxygen saturation and thereby tracks SvO_2_ across distinct experimental shock paradigms. These preclinical findings support further investigation of esophageal RRS as a potential minimally invasive monitoring approach for detection of systemic oxygen imbalance.

**Supplementary Information:**

The online version contains supplementary material available at https://doi.org/10.1186/s40635-026-00989-z.

## Introduction

Early recognition of inadequate tissue oxygenation is essential in the management of critically ill patients [1, 2]. Mixed venous oxygen saturation (SvO_2_) is an integrative marker of the balance between systemic oxygen delivery (DO_2_) and oxygen consumption (VO_2_) [2]. It has established value in the assessment of shock states [3, 4], cardiac output adequacy, and systemic oxygenation beyond conventional vital signs or arterial measurements [5]. However, measuring SvO_2_ requires pulmonary artery (PA) catheterization or central venous access, which carries risks such as infection, thrombosis, and vascular injury [6], and is generally limited to patients with independent indications for invasive monitoring [5, 7].

Near-infrared spectroscopy (NIRS) is widely used to estimate regional tissue oxygenation through differential light absorbance and has also been studied in the context of shock monitoring [8, 9]. It integrates signals from spectrophotometrically active arterial, venous, and intracellular structures, including myoglobin [10], and features deep tissue penetration, yielding a composite regional oxygenation index rather than a discrete measure of venous hemoglobin oxygen saturation [11]. Its accuracy is limited by assumptions of static arterial–venous blood volume ratios [12] and myoglobin contamination (which remains oxygenated in all but the most extremes of tissue hypoxia); this limits detection of critical hypoxia [13]. More recent research approaches compute estimates of SvO_2_ based on measurements of VO_2_, cardiac output, and arterial saturation (SaO_2_) [14], however, these approaches are technically complex and impractical for rapid bedside decision-making [14, 15], highlighting the need for noninvasive, continuous monitoring methods.

In contrast to absorbance-based methods, resonance Raman spectroscopy (RRS) offers molecularly specific detection of hemoglobin vibrational signatures [16]. Raman scattering arises from inelastic photon–molecule interactions, in which energy is transferred to molecular vibrational modes, producing a measurable wavelength shift characteristic of specific molecular bonds. Tuning the wavelength near the Soret absorption band (~ 400–450 nm) selectively amplifies scattering from the heme porphyrin ring [17], yielding a strong hemoglobin signal and allowing distinction from other tissue chromophores, including myoglobin [16, 18–20]. Raman-based assessment of mitochondrial redox state has been proposed as a noninvasive method to evaluate tissue oxygen delivery and mitochondrial function in vivo, with potential applications in monitoring tissue viability, ischemia, and organ preservation [19].

Leveraging the molecular specificity of Raman spectroscopy, we previously described a miniaturized RRS probe fitted into the retropharyngeal space that predicted time to death in a model of lethal hemorrhage [20]. Here, we evaluate a novel designed esophageal RRS probe which measures regional capillary hemoglobin oxygen saturation (S_Es_O_2_). We hypothesize that S_Es_O_2_ tracks SvO_2_ across shock states and thereby enables minimally invasive detection of systemic oxygen imbalance.

## Materials and methods

### Ethical approval and animal care

All experimental procedures were approved by the Institutional Animal Care and Use Committee (IACUC protocol 00002467) at Boston Children’s Hospital and performed in compliance with the National Institutes of Health *Guide for the Care and Use of Laboratory Animals*. Male Sprague Dawley rats (400–500 g) were obtained from Charles River Laboratories (Wilmington, MA, USA) and housed in pairs under controlled environmental conditions (temperature 21–22 °C; humidity 40–50%) with a 12-h light–dark cycle and ad libitum access to food and water. Animals were acclimated for at least 7 days prior to experimentation. This study is reported in accordance with the ARRIVE 2.0 guidelines (Supplemental 1).

### Anesthesia, airway management, and ventilation

On the day of experimentation, anesthesia was induced with inhaled isoflurane (2.5%) in an induction chamber. After loss of righting reflex, animals received intraperitoneal ketamine (45–75 mg/kg) and xylazine (5–10 mg/kg) for sustained sedation and analgesia. Animals were intubated with a 14-gauge, 2-inch catheter under direct visualization and mechanically ventilated (SAR–1000 ventilator, CWE Inc., Ardmore, PA, USA) at tidal volume 8–10 mL/kg, respiratory rate (35–45/min), positive end-expiratory pressure 5 cm H_2_O, and fraction of inspired oxygen (FiO_2_) set to 30–35% at baseline. Anesthesia was titrated with 1–2% isoflurane and (rectal) body temperature was maintained at 37 °C using a closed-loop thermoregulated heating pad.

### Instrumentation

After induction, one femoral artery was cannulated with a Millar SPR-671 (1.4F) catheter for continuous pressure monitoring, and the contralateral femoral artery was cannulated (24G needle) for arterial blood sampling. Mixed venous blood was collected via thoracotomy and opening of the pericardium followed by direct puncture of the pulmonary artery (26G needle) [20]. Sample volumes were 0.1–0.2 mL, representing minimal blood loss (30 mL estimated circulating volume in a 500-g rat), and thus were not replaced. Bleeding was controlled with a cotton swab, and no hemodynamic changes were observed during sampling. All blood gas measurements were immediately analyzed (*ABL90 Flex Plus, Radiometer U.S.A.).* Transabdominal NIRS (*Somanetics Invos 5100C*) was positioned on the abdominal wall over the left upper quadrant and secured with adhesive tape. To prevent signal interference, all rats were shaved prior to sensor placement and the abdominal area was cleaned with medical-grade ethanol wipes. After instrumentation, animals were allowed to stabilize for 20 min prior to baseline data acquisition.

### Resonance Raman spectroscopy system

**Fig. 1:**
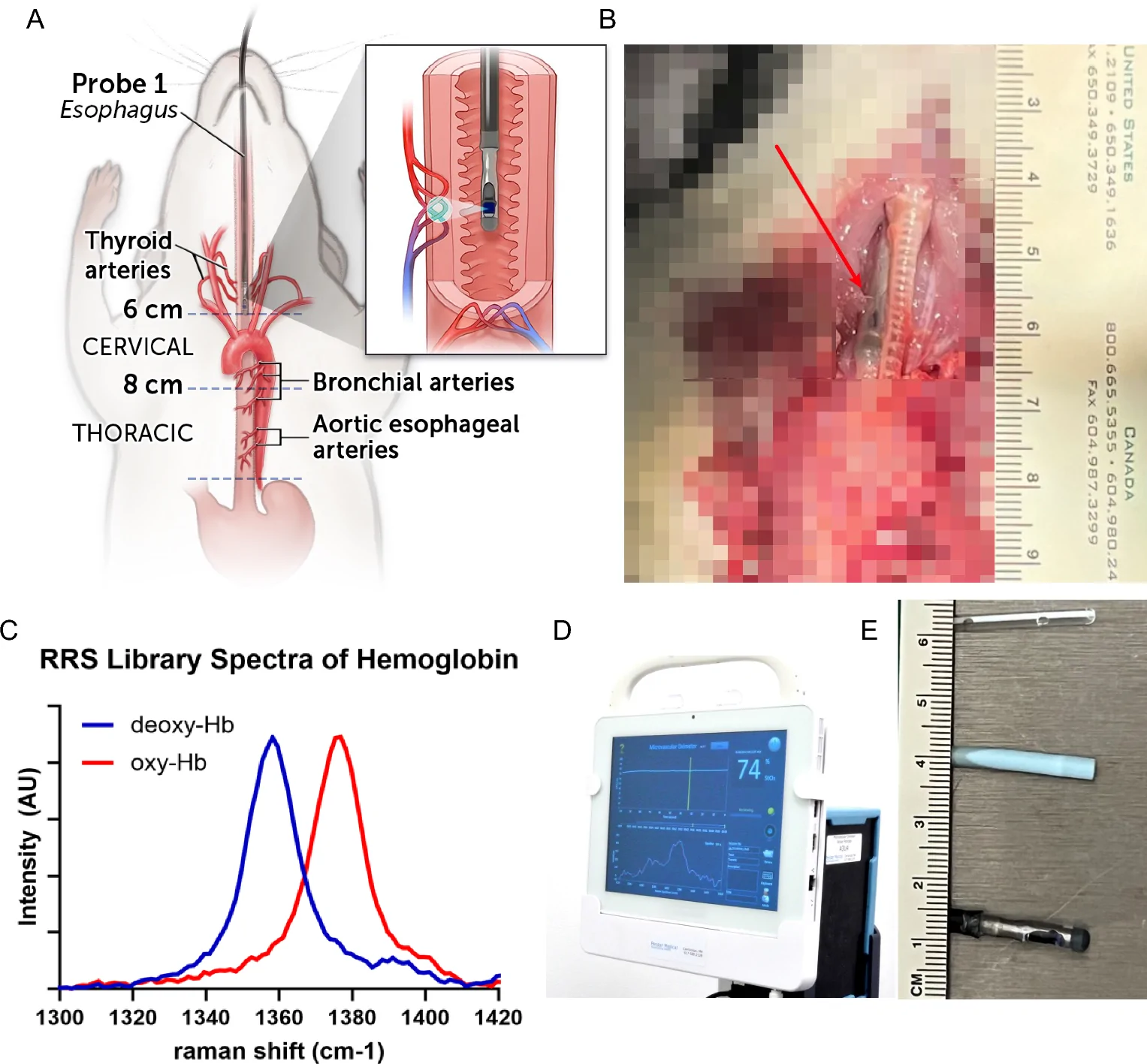
Esophageal Resonance Raman Spectroscopy (RRS) instrumentation. **A**) Schematic of probe placement: cervical (6 cm) and thoracic (8+ cm), call out brackets represent blood supply from thyroid and bronchial/aortic arteries. **B**) Overview of probe placement during experiments. **C**) RRS library spectra of oxy- (red) and deoxy-hemoglobin (blue). **D**) Portable Raman bedside system tablet interface. **E**) Fiber-optic RRS probe (bottom) vs. standard 8F feeding/aspiration tubes (middle, top, ruler = in cm)

We further developed a previously described RRS system [19, 20] to incorporate optical fibers into a flexible 4 mm diameter linear probe designed for insertion into the esophagus. The probe was advanced into the proximal cervical esophagus under direct visualization (Fig. 1A-B). The system uses 405 nm excitation near the Soret band, enabling resonance enhancement of porphyrin vibrational modes and discrimination between oxy- and deoxyhemoglobin via the ν4 Raman band (Fig. 1C). Results were displayed on a portable bedside system (Fig. 1D) and averaged per 60 s for analysis.

### Signal processing and saturation calculation

Spectral decomposition was performed using a regression-based algorithm with reference spectra for oxy-hemoglobin and deoxy-hemoglobin. The relative contributions of each spectral component were calculated using least-squares fitting. Percent hemoglobin oxygen saturation (S_Es_O_2_) was computed as:

$${S}_{Es}{O}_{2}(\%) =\frac{\left[Hb{O}_{2}\right]}{\left[Hb{O}_{2}\right]+\left[HHb\right]}\times 100\%$$

All spectral processing was performed using custom software written in Python.

### Experimental protocols

#### Progressive hemorrhagic shock (n=12)

**Fig. 2:**
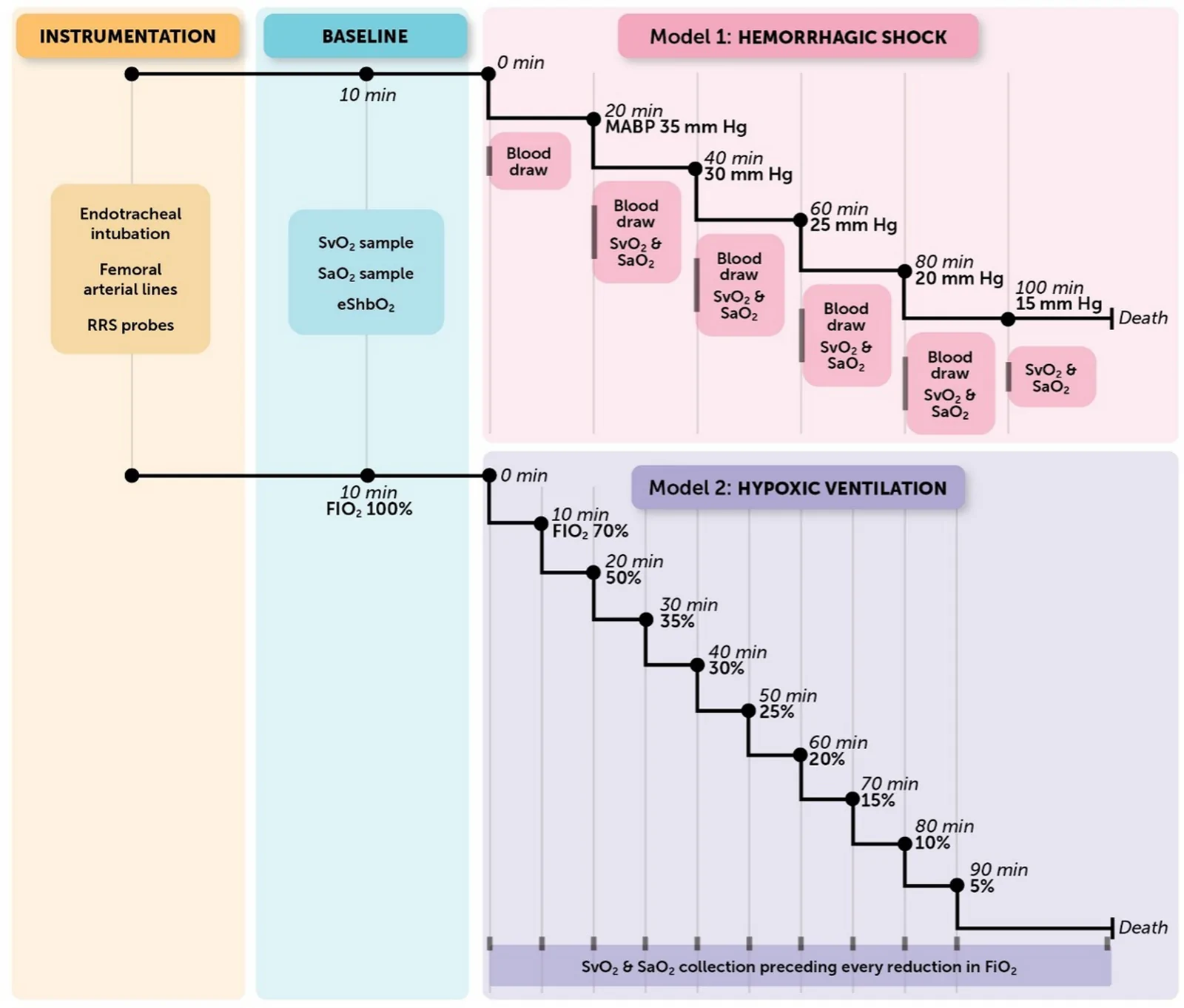
Experimental timelines for progressive hemorrhage and hypoxic ventilation protocols. Model 1: Hemorrhage (n = 12): MABP reduced via phlebotomy in 5 mmHg steps to a terminal level of 15 mmHg; each stage was maintained 20 min before sampling of SvO_2_ and lactate. Model 2: Hypoxia (n = 9): FiO_2_ was reduced stepwise from 100 to 5%. Each stage was maintained for 10 min. Shaded areas indicate the 2-min steady-state windows used for data acquisition prior to each sampling stage

After a 10-min baseline period, controlled phlebotomy was performed via the femoral arterial catheter to reduce mean arterial blood pressure (MABP) to 35 mmHg, designated as timepoint 0. MABP was then sequentially reduced to 30, 25, 20, and 15 mmHg. Each target MABP was maintained for 20 min before sampling by withdrawing small aliquots of blood or administering saline, after which the pulmonary artery (PA) was sampled for SvO_2_ and lactate analysis. Hemorrhage was continued until MABP reached 15 mmHg or until death, defined as pulse pressure (systolic–diastolic pressure) <5 mmHg. Hemodynamic parameters, transabdominal NIRS and S_Es_O_2_ were recorded continuously. For analyses, S_Es_O_2_ values were averaged over the 2 min immediately preceding each SvO_2_ and lactate sample (Fig. 2).

#### Hypoxic ventilation model (n=9)

After baseline stabilization at 100% FiO_2_, FiO_2_ was reduced stepwise to 70%, 50%, 35%, 30%, 25%, 20%, 15%, 10%, and 5%. Each FiO_2_ level was maintained for 10 min prior to arterial and pulmonary artery sampling. Hemodynamic parameters, transabdominal NIRS and S_Es_O_2_ were recorded continuously. Like in the hemorrhage model, S_Es_O_2_ values were averaged over the 2 min preceding each SvO_2_ measurement for analysis (Fig. 2).

### Statistical analysis

Statistical analyses were performed in Prism (GraphPad Software, CA, USA) and Python (v.3.9). Continuous variables were reported as mean ± SEM and two-sided p < 0.05 was considered significant. Per-stage changes in the hemorrhage and hypoxia models were evaluated with one-way repeated-measures ANOVA and Holm-Sidak post-hoc analysis. Separate sensitivity analyses for hemorrhage were conducted by repeating primary analysis for stages with 10 animals (MABP ≥ 25 mmHg) and comparison of baseline (MABP ≈ 60 mmHg) values between completers vs non-completers of the protocol by Welch’s t-test. The primary outcome (S_Es_O_2_–SvO_2_ relationship) was analyzed using linear mixed-effects (LME) models, with S_Es_O_2_ as a fixed effect and animal identity as a random intercept; all available observations were included. Model assumptions were assessed visually with residuals-versus-fitted and Q–Q plots (Supplemental 2). An analogous LME model was fit for lactate, with S_Es_O_2_ as the fixed effect. For transabdominal NIRS, an LME model was fit with NIRS as the fixed effect and S_Es_O_2_ as the outcome, again with animal identity as a random intercept. Pearson correlation coefficients are also reported as descriptive summaries of bivariate associations of other recorded parameters.

A formal agreement analysis (e.g., Bland–Altman) was not performed because S_Es_O_2_ and SvO_2_ sample distinct physiological compartments (regional esophageal capillary versus global mixed-venous blood) with an expected offset, meaning absolute agreement is not expected. Consistency of tracking across animals is visualized by within-animal trajectory plots (Supplemental 3). The diagnostic performance for severe oxygen deficit (SvO_2_ < 40%, based on established associations with anaerobic metabolism and adverse clinical outcomes [21]) was assessed at the animal level using cluster bootstrap receiver operating characteristic (ROC) analysis with 2,000 resampling iterations. Optimal cut-offs were identified using Youden’s index, and corresponding sensitivity and specificity are reported (Supplemental 5).

## Results

### Animal preparation and protocol completion

**Fig. 3:**
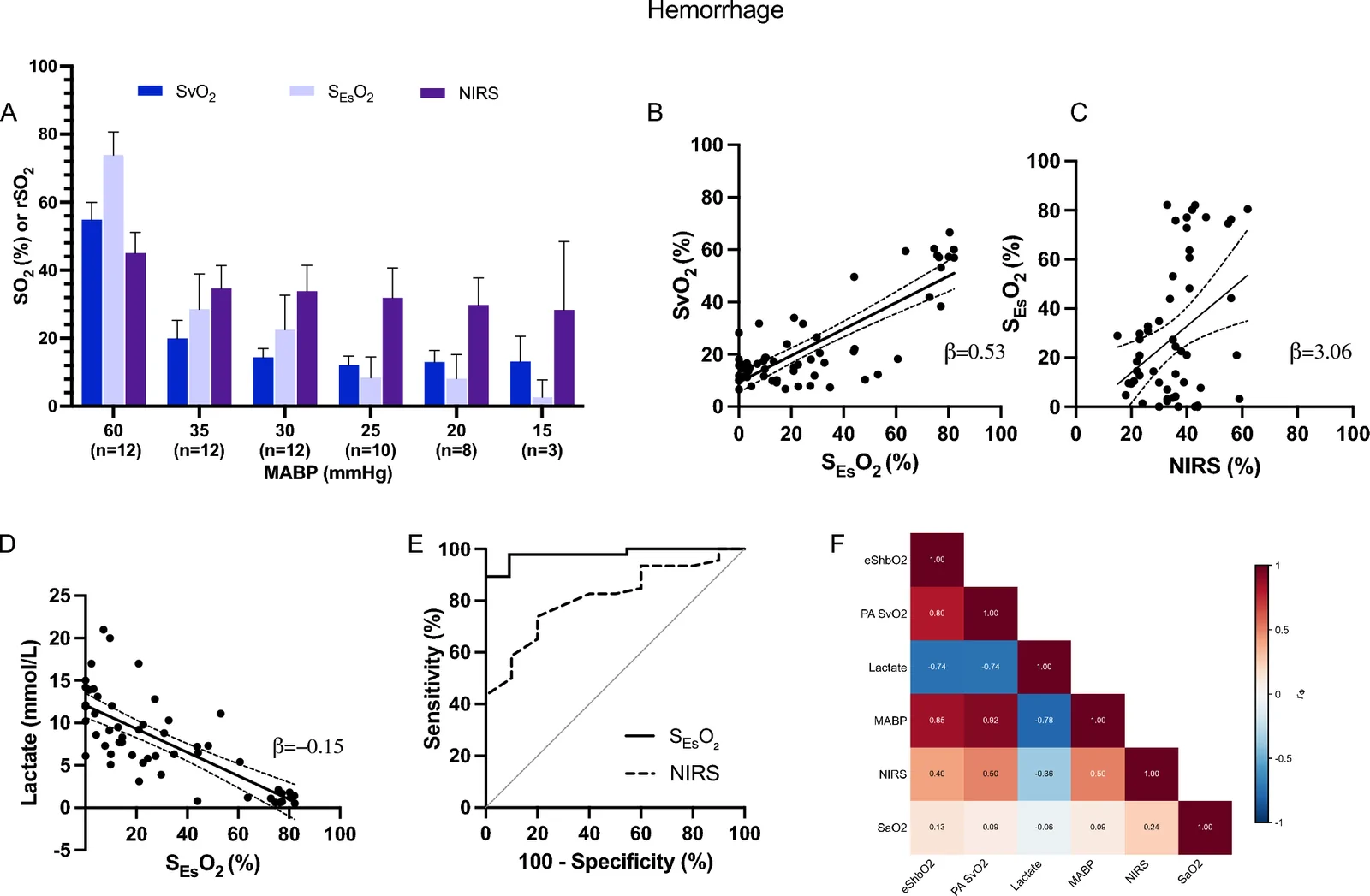
Oxygen measurements in progressive hemorrhage model of Male Sprague Dawley rats (n = 12). Rats were subjected to phlebotomy to reduce MABP from baseline to 35, 30, 25, 20, and 15 mmHg. SvO_2_, S_Es_O₂, transabdominal NIRS and MABP were continuously sampled and lactate and SaO_2_ were sampled after every stage. **A**) Average SvO_2_, S_Es_O_2_, and transabdominal NIRS per MABP stage. Data are mean ± SEM; number of animals completing stage indicated (n). **B**) Association of S_Es_O₂ vs. SvO_2_. **C**) Association of S_Es_O_2_ vs transabdominal NIRS. **D**) Association of Lactate vs S_Es_O_2_ **E**) ROC curves for detection of severe oxygen deficit (SvO_2_ < 40%). **F**) Pearson correlation matrix. Number of animals completing each stage is indicated on the x-axis (n). Lines are LME slope with 95% CI (**B-D**)

**Fig. 4:**
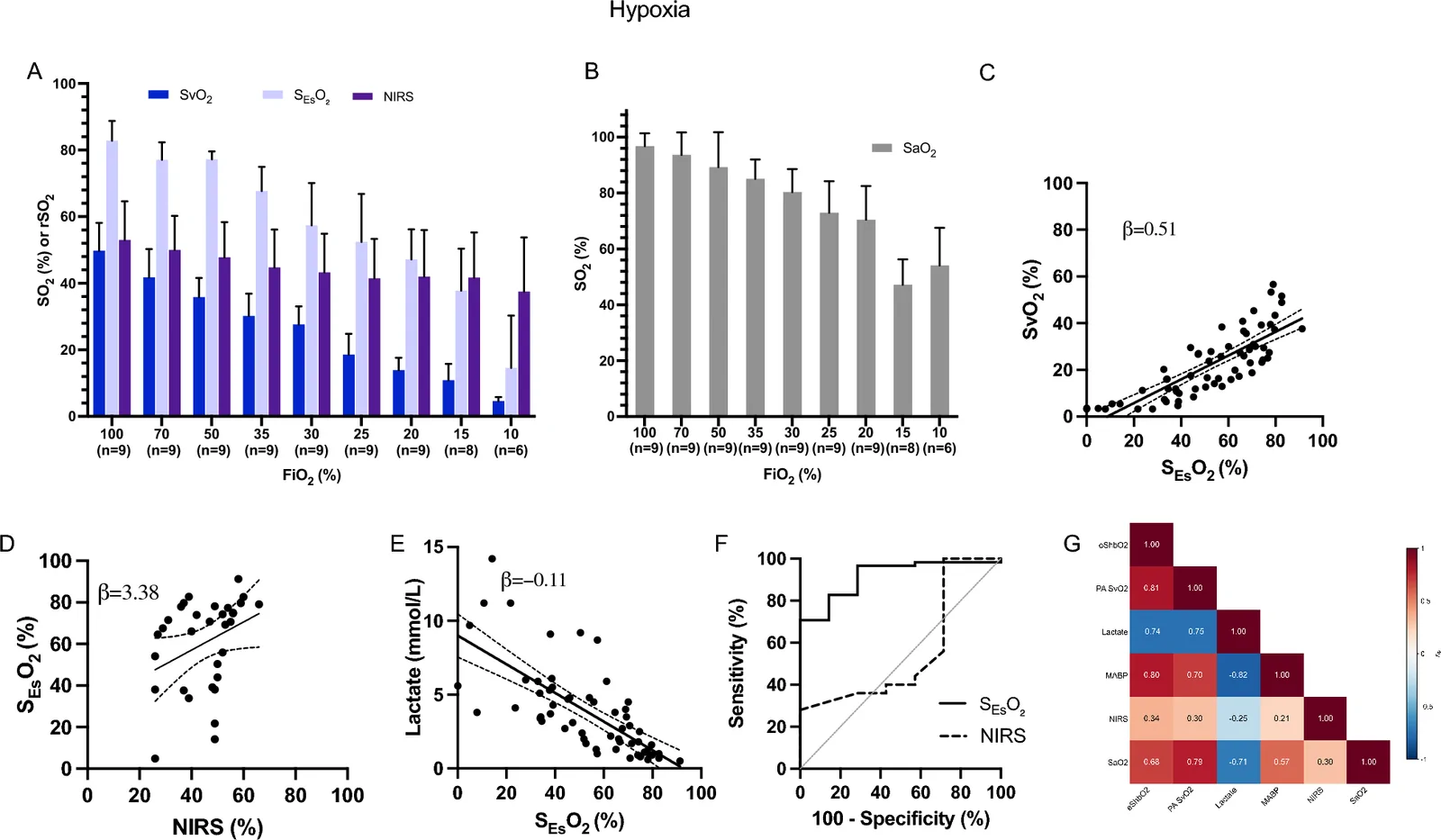
Oxygenation and metabolic responses during progressive hypoxic ventilation in Male Sprague Dawley rats (n = 9). Rats were subjected to stepwise reductions in FiO_2_ from baseline (100%) to 5% to induce graded systemic hypoxia. S_Es_O_2,_ MABP, transabdominal NIRS were continuously monitored, lactate, SaO_2_ ​and SvO_2_ were sampled at the conclusion of each stage. **A**) Average SvO_2_, S_Es_O_2,_ and transabdominal NIRS per FiO₂ stage; number of animals completing each stage is indicated (n). Data are presented as mean ± SEM. **B**) Average SaO_2_ measured at each FiO_2_ stage. **C**) Association of S_Es_O_2_ versus SvO_2_. **D**) Association of S_Es_O_2_ versus transabdominal NIRS. **E**) Association of Lactate versus S_Es_O_2_. **F**) ROC curves for detection of severe oxygen deficit (SvO_2_ < 40%). **G**) Pearson correlation matrix. Lines are LME slope with 95% CI (C-E). Number of animals completing each stage is indicated on the x-axis (n)

Progressive hemorrhage was initiated in 12 animals and 3 animals survived down to MABP 15 mmHg, 8 to MABP 20 mmHg, and 10 to MABP 25 mmHg, respectively. The baseline S_Es_O_2_ and SvO_2_ did not differ significantly between animals completing the full protocol (n = 3) and those that did not (n = 9). The baseline S_Es_O_2_ was 72.4 ± 7.6% vs. 74.4 ± 11.9% (p = 0.75, Welch’s t-test), baseline SvO_2_ 56.6 ± 3.1% vs. 54.3 ± 9.2% (p = 0.54) and baseline lactate 0.8 ± 0.3 vs. 1.2 ± 0.6 mmol/L (p = 0.16) were not significantly different, suggesting that early mortality was related to individual hemodynamic tolerance rather than systematic baseline differences. There was no mortality during instrumentation phase. Hypoxic ventilation was initiated in 9 animals and completed to FiO_2_ of 10% in 6 animals. Intra-experimental survival is presented per-stage on the x-axis of Figs. 3 and 4. Across both experimental paradigms, baseline S_Es_O_2_ values exceeded pulmonary artery SvO_2_ by approximately 18–22%, consistent with a regional capillary measurement distinct from SvO_2_.

### Progressive hemorrhagic shock

#### Hemodynamic and oxygenation responses

Controlled phlebotomy resulted in stepwise reductions of MABP from baseline 60 ± 0.0 mmHg to 15.1 ± 0.4 mmHg (p < 0.0001). SvO_2_ decreased from 53.8 ± 2.1% at baseline to 12.4 ± 1.5% at the final hemorrhage stage 15 mmHg (p < 0.0001). S_Es_O_2_ decreased from 72.1 ± 2.8% at baseline to 3.1 ± 0.6% at MABP 15 mmHg (p < 0.0001). SaO_2_ remained within normal physiological range throughout hemorrhage (baseline 99.4 ± 0.2% vs 99.6 ± 0.1% at final stage, p = 0.042). Although RM-ANOVA yielded a nominally significant p-value (p = 0.042), the absolute change was clinically negligible (< 1%).

Transabdominal NIRS measurements changed mildly with increasing severity of shock (45.0 ± 2.7% at baseline to 29.8 ± 3.4% at MABP 15 mmHg). Serum lactate increased from 1.1 ± 0.2 mmol/L at baseline to 8.4 ± 1.1 mmol/L at MABP 15 mmHg (p < 0.0001), indicating a metabolic response to shock. One-way RM-ANOVA demonstrated overall significance of experimental conditions on all physiological variables except transabdominal NIRS (p < 0.05 for all), with differences from baseline apparent at the initial stage (35–30 mmHg, Holm–Sidak) (Fig. 3A).

#### Associations of S_Es_O_2_ with SvO_2_, NIRS, and lactate

In the hemorrhage model, S_Es_O_2_ was positively associated with SvO_2_ (Fig. 3B), corresponding to a 0.53 percentage point increase in SvO_2_ for each 1 percentage point increase in S_Es_O_2_ (β = 0.53, 95% CI 0.43–0.62; p < 0.0001) based on 58 observations from 12 animals in a linear mixed-effects model with animal as a random intercept. S_Es_O_2_ was also positively associated with transabdominal NIRS (Fig. 3C), corresponding to a 3.06 percentage point increase in S_Es_O_2_ for each 1 percentage point increase in NIRS (β = 3.06, 95% CI 2.09–4.02; p < 0.0001) based on 55 observations from 11 animals, although substantial scatter was observed. S_Es_O_2_ was inversely associated with lactate (Fig. 3D), corresponding to a 0.15 mmol/L decrease in lactate for each 1 percentage point increase in S_Es_O_2_ (β = −0.15, 95% CI −0.18 to −0.12; p < 0.0001), based on 53 observations from 12 animals.

#### Diagnostic performance

Severe oxygen deficit was defined a priori as SvO_2_ <40% for ROC analysis. S_Es_O_2_ discriminated this state with an AUC of 0.98 (95% CI: 0.95–1.00; 2,000 iterations resampled at the animal level; n = 58 observations). The optimal S_Es_O_2_ cutoff identified by Youden’s index was < 43.9%, yielding 89.4% sensitivity, 100% specificity, 100% PPV, and 68.8% NPV. Transabdominal NIRS yielded an AUC of 0.82 (95% CI 0.70 – 0.95, p = 0.0021; n = 56 observations). (Fig. 3E, supplemental 5).

#### Correlation analysis

S_Es_O_2_ demonstrated strong positive correlations with SvO_2_ (r = 0.81, p < 0.0001) and MABP (r = 0.86, p < 0.0001), alongside a robust inverse association with serum lactate (r = − 0.74, p < 0.0001). There was no apparent correlation between S_Es_O_2_ and SaO_2_ (r = 0.13, p = 0.33) (Fig. 3F).

### Hypoxic ventilation model

#### Oxygenation responses

Stepwise reduction in FiO_2_ resulted in proportional decreases in arterial and venous oxygen saturation. SvO_2_ decreased from 49.8 ± 4.2% at baseline to 4.6 ± 0.5% at the lowest FiO_2_ (p < 0.001). S_Es_O_2_ decreased from 82.8 ± 3.0% to 14.6 ± 5.9% across the protocol (p < 0.001). Serum lactate increased progressively from 0.68 ± 0.09 mmol/L to 8.6 ± 1.5 mmol/L (p < 0.001). Both S_Es_O_2_ and SvO_2_ showed significant divergence from baseline at the earliest titration stage, whereas transabdominal NIRS did not reach significance (p = 0.057) (Fig. 4A). Average SaO_2_ measured invasively in the femoral artery was 90.9 ± 8.2% and dropped down to 47.1 ± 14.2% at the lowest stage of hypoxia reached in each rat (Fig. 4B).

#### Associations of S_Es_O_2_ with SvO_2_, NIRS, and lactate

In the hypoxia model, S_Es_O_2_ was positively associated with SvO_2_ (Fig. 4C), corresponding to a 0.51 percentage-point increase in SvO_2_ for each 1-percentage point increase in S_Es_O_2_ (β = 0.51, 95% CI 0.43–0.60; p < 0.0001), based on 65 observations from 9 animals. S_Es_O_2_ was also positively associated with transabdominal NIRS (Fig. 4D), corresponding to a 3.38 percentage point increase in S_Es_O_2_ for each 1-percentage point increase in NIRS (β = 3.38, 95% CI 2.17–4.58; p < 0.0001), based on 32 observations from 4 animals, with substantial scatter present. S_Es_O_2_ was inversely associated with lactate (Fig. 4E), corresponding to a 0.11 mmol/L decrease in lactate for each 1-percentage point increase in S_Es_O_2_ (β = −0.11, 95% CI −0.13 to −0.09; p < 0.0001), based on 59 observations from 8 animals.

#### Diagnostic performance

Using the a priori threshold of SvO_2_ < 40%, S_Es_O2 identified severe oxygen deficit with an AUC of 0.92 (95% CI: 0.82–1.00; n = 65 observations from 9 animals). The optimal S_Es_O_2_ cutoff was < 65.0% (Youden’s index), yielding 70.7% sensitivity, 100% specificity, 100% PPV, and 29.2% NPV. Transabdominal NIRS showed limited ability to detect severe hypoxia (AUC 0.56, 95% CI: 0.23–1.00; n = 32, available for n = 4 animals) (Fig. 4F, Supplemental 5).

#### Correlation analysis

S_Es_O_2_ and SvO_2_ demonstrated strong Pearson correlation (r = 0.82, p < 0.0001). S_Es_O_2_ demonstrated positive correlation with MABP (r = 0.82, p < 0.0001) and inverse correlation with serum lactate (r = −0.71, p < 0.0001). S_Es_O_2_​ also exhibited a significant positive correlation with arterial saturation (SaO_2_, r = 0.68, p < 0.0001), accurately reflecting the systemic desaturation inherent to this experimental protocol (Fig. 4G).

### Sensitivity analysis for hemorrhage model MABP ≥ 25 mmHg

To evaluate whether the primary findings were driven by the small number of animals surviving to the most severe hemorrhage stages, all analyses were repeated using only stages at which ≥10 animals contributed data (MABP ≥ 25 mmHg; n = 48 observations from 12 animals). S_Es_O_2_ remained strongly correlated with SvO_2_ (r = 0.79, p < 0.0001). The LME slope estimate was β = 0.56 (95% CI 0.45–0.67, p < 0.0001), consistent with the full model estimate (β = 0.53). ROC analysis yielded an AUC of 0.98 (95% CI: 0.93–1.00) for detection of SvO_2_ < 40%, with an optimal S_Es_O_2_ cutoff of < 60.7% (sensitivity 97.3%, specificity 90.9%). These results confirm that the observed associations are not driven by the limited number of animals at the most severe stages.

## Discussion

We have shown that S_Es_O_2_ tracks changes in SvO_2_ across two physiologically distinct models of systemic oxygen deficit. In both hemorrhagic and hypoxic shock, S_Es_O_2_ showed strong association with SvO_2_ and inverse association with serum lactate, supporting its relevance as a marker of impaired oxygen delivery-consumption balance. In addition, S_Es_O_2_ demonstrated high diagnostic accuracy for identifying severe oxygen deficit, defined as SvO_2_ < 40%. Together, these findings suggest that esophageal Raman spectroscopy provides a continuous, minimally invasive measurement of esophageal capillary hemoglobin saturation that reflects physiologically meaningful changes in systemic oxygen balance.

A key implication of our findings is that the primary value of esophageal Raman spectroscopy lies in continuous trend monitoring rather than point estimation of SvO_2_. At baseline, S_Es_O_2_ was approximately 20 percentage points higher than SvO_2_, consistent with measurement in a regional esophageal capillary bed rather than in true mixed venous blood. This offset was not maintained across all stages. During severe hemorrhage, S_Es_O_2_ fell to below SvO_2_. Its clinical value therefore lies not in absolute agreement with SvO_2_ (it is not a direct substitute) and more in longitudinal tracking of oxygen imbalance. In many clinical settings, intermittent measurements of central venous saturation are used to guide resuscitation, but these approaches are limited by their episodic nature and dependence on invasive access. In contrast, S_Es_O_2_ provides a continuous signal that reflects evolving changes in oxygen delivery and consumption. In both models, S_Es_O_2_ declined in parallel with worsening physiologic stress and increasing lactate, suggesting that it captures early deterioration in systemic oxygen balance.

The observed differences in performance between S_Es_O_2_ and transabdominal NIRS are likely explained by their underlying measurement principles and the NIRS application used here. NIRS relies on absorbance-based methods that generate a composite signal influenced by arterial, venous, and intracellular chromophores, including myoglobin, and require assumptions regarding vascular compartment contributions [13]. These factors have been shown to limit sensitivity to extreme tissue hypoxia, whether by trend or point measurement, particularly in severe shock states [13, 22]. In contrast, RRS selectively interrogates hemoglobin vibrational structure and directly distinguishes oxyhemoglobin from deoxyhemoglobin. This molecular specificity reduces contamination from non-hemoglobin chromophores and may preserve sensitivity across a wider range of oxygenation states. Consistent with this mechanism, S_Es_O_2_ demonstrated strong associations with SvO_2,_ and numerically higher discrimination of severe oxygen deficit compared to transabdominal NIRS in both models, although in the hypoxia model NIRS was available for only four animals. The Youden cut-offs and the corresponding sensitivity, specificity, PPV, and NPV were derived and evaluated in the same small datasets. These estimates are exploratory and may be optimistic and require independent validation. Importantly, PPV and NPV also depend on the experimentally generated prevalence of SvO_2_ < 40%. Lastly, because NIRS was applied transabdominally in this rodent model, the resulting measurements may differ from clinical cerebral NIRS signals, which limits direct generalizability of these findings.

The esophagus represents a practical and physiologically relevant site for monitoring. It is centrally located, readily accessible, and relatively shielded from external optical interference. The esophageal microcirculation is also subject to dynamic redistribution during shock, which may enhance sensitivity to changes in systemic oxygen delivery. These features, combined with the established safety profile of esophageal instrumentation for thermometry and echocardiography, support its potential clinical feasibility [23]. Importantly, the device design may allow integration into existing clinical tools, such as enteric feeding tubes or temperature probes. This may be particularly relevant in patient populations where invasive monitoring is undesirable or impractical, including pediatric patients, individuals with limited vascular access, and patients in whom the risks of pulmonary artery or central venous catheterization outweigh the potential benefits. Future work should prioritize further pre-clinical testing of probe performance during vasoactive hemodynamic support, long-term safety, and efficacy in less-severe models of shock [24]. In parallel, device-focused development should optimize a human-scale, sterilizable probe with practical handling features.

Several limitations warrant discussion. First, the comparative transabdominal NIRS measurements were conducted on the anterior abdominal wall of a rodent. Although there is precedent for this approach [25, 26], it does differ from the more common clinical applications of NIRS on the forehead and/or flank. Second, factors such as (long-term) probe positioning, mucosal integrity, secretions, and motion may influence signal quality. Systemic factors, including anemia, vasoactive medications, and alterations in regional blood flow, may also affect the relationship between S_Es_O_2_ and global oxygenation. Third, a formal agreement analysis was not performed, as S_Es_O_2_ and SvO_2_ reflect distinct physiological compartments and measurement techniques; accordingly, analyses prioritized the evaluation of dynamic tracking and temporal correlation rather than absolute interchangeability. Fourth, the effective sample size decreased at the most severe stages of both models because mortality is inherent to lethal shock protocols. While in additional analyses to account for attrition bias similar trends were observed, these sparse data at the most extreme stages may still limit generalizability. Fifth, diagnostic performance was strongest in advanced shock. Whether S_Es_O_2_ can effectively detect early imbalance in less severe models requires further investigation.

## Conclusions

Esophageal RRS offers minimally invasive, molecularly specific measurement of regional capillary hemoglobin saturation that tracks mixed venous oxygenation in diverse shock states. These preclinical findings support further investigation of esophageal RRS as a potential minimally invasive approach for continuous monitoring of systemic oxygen imbalance in critically ill patients.

## Supplementary Information

Supplementary Material 1.

Supplementary Material 2.

## Data Availability

The datasets generated and analyzed during the current study are available from the corresponding author upon reasonable request.

## Abbreviations

SvO_2_: (Pulmonary artery sampled) mixed venous oxygen saturation
S_Es_O_2_: Esophageal regional capillary hemoglobin saturation (esophageal tissue oxygenation)
RRS: Resonance Raman spectroscopy
SaO_2_: Arterial oxygen saturation
NIRS: Near-infrared spectroscopy
OLS: Ordinary least squares
ROC: Receiver operating characteristic (curve)
AUC: Area under the curve
DO_2_: Oxygen delivery
VO_2_: Oxygen consumption
PA: Pulmonary artery
IACUC: Institutional animal care and use committee
FiO_2_: Fraction of inspired oxygen
MABP: Mean arterial blood pressure
RM-ANOVA: Repeated-measures analysis of variance
LME: Linear mixed-effects (model)
SEM: Standard error of the mean
HbO_2_: Oxyhemoglobin
HHb: Deoxyhemoglobin (deoxygenated hemoglobin)
