# Supplementary material for: Esophageal resonance Raman spectroscopy tracks mixed venous oxygenation in experimental shock and hypoxemia
Source: Intensive Care Med Exp. 2026 Oct 15;14:138. doi: 10.1186/s40635-026-00989-z (PMC13672757; doi:10.1186/s40635-026-00989-z)
Supplement: Supplementary file 2 [file 40635_2026_989_MOESM2_ESM.pdf]

Supplemental 2. Residual Diagnostics of Linear Mixed Effects Models for $S_{Es}O_2$, Lactate and SvO_2 relationships.

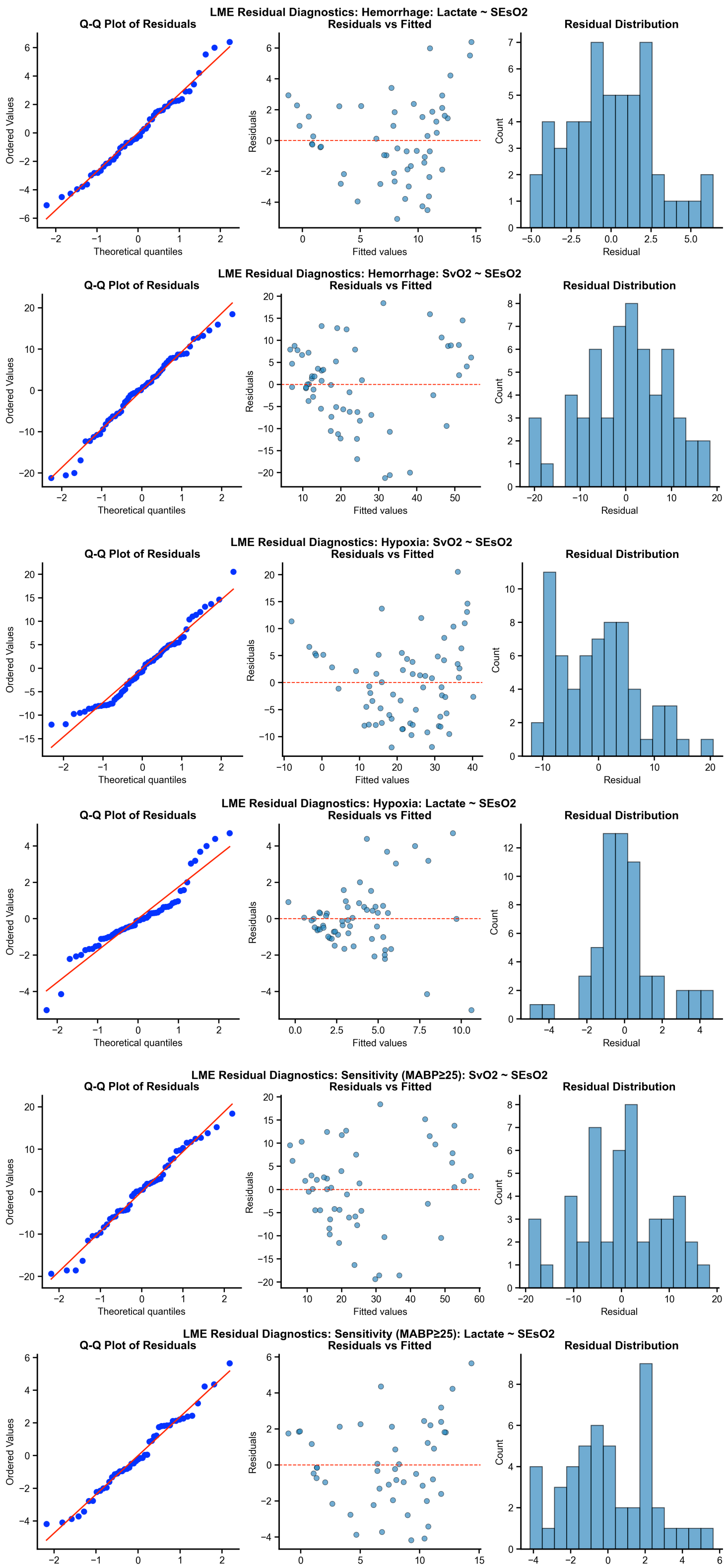

Supplemental 3. Spaghetti plots of within-animal trajectories of SvO_2 and $SEsO_2$

A) Hemorrhage Shock Model

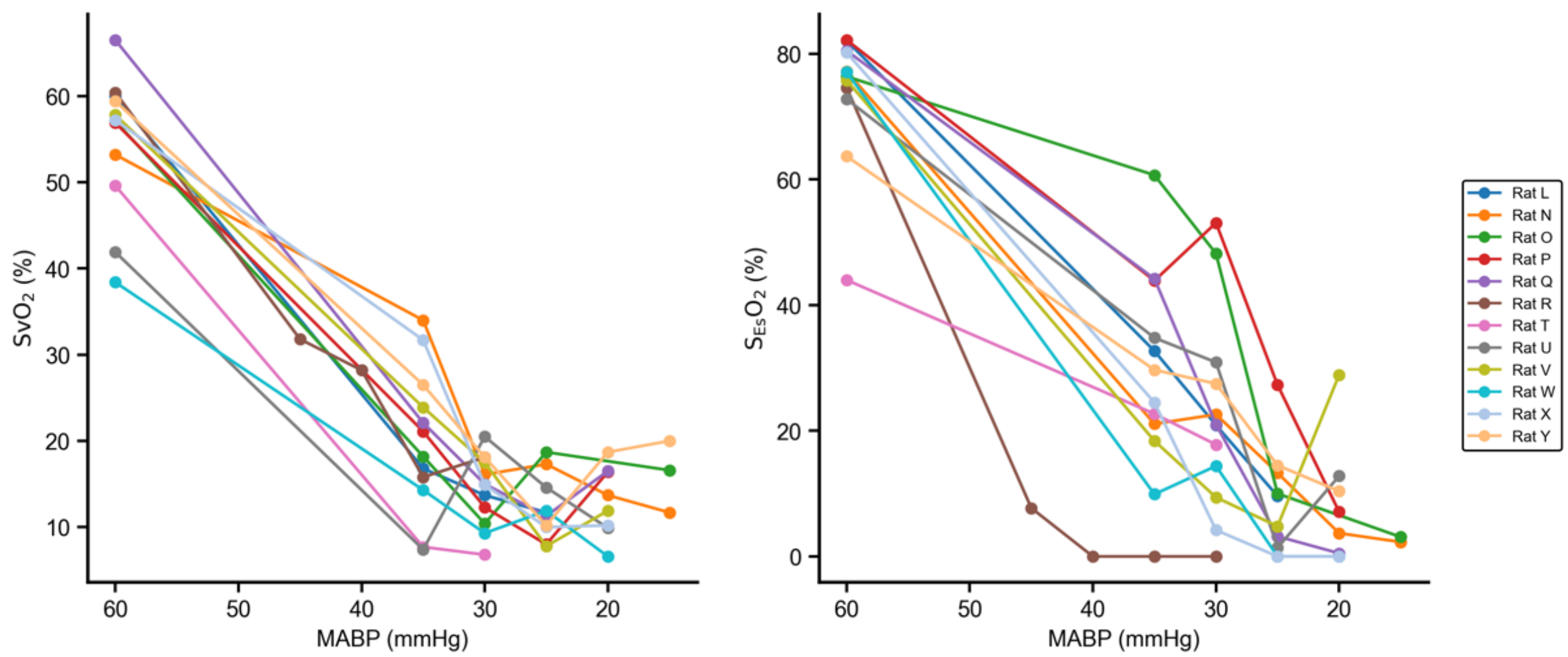

B) Hypoxic Shock Model

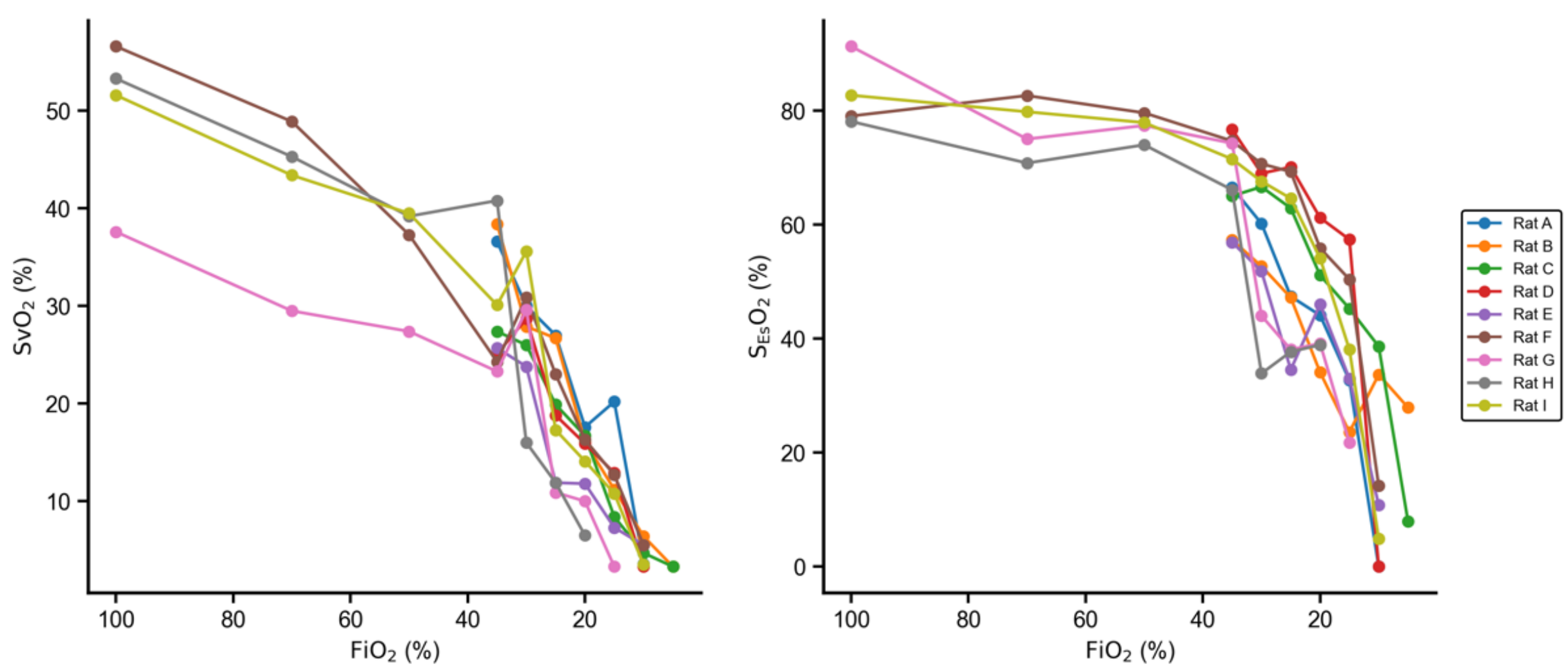

Supplemental 4. Colored correlation plots for visualization of per-animal contributions.

A) Hemorrhage

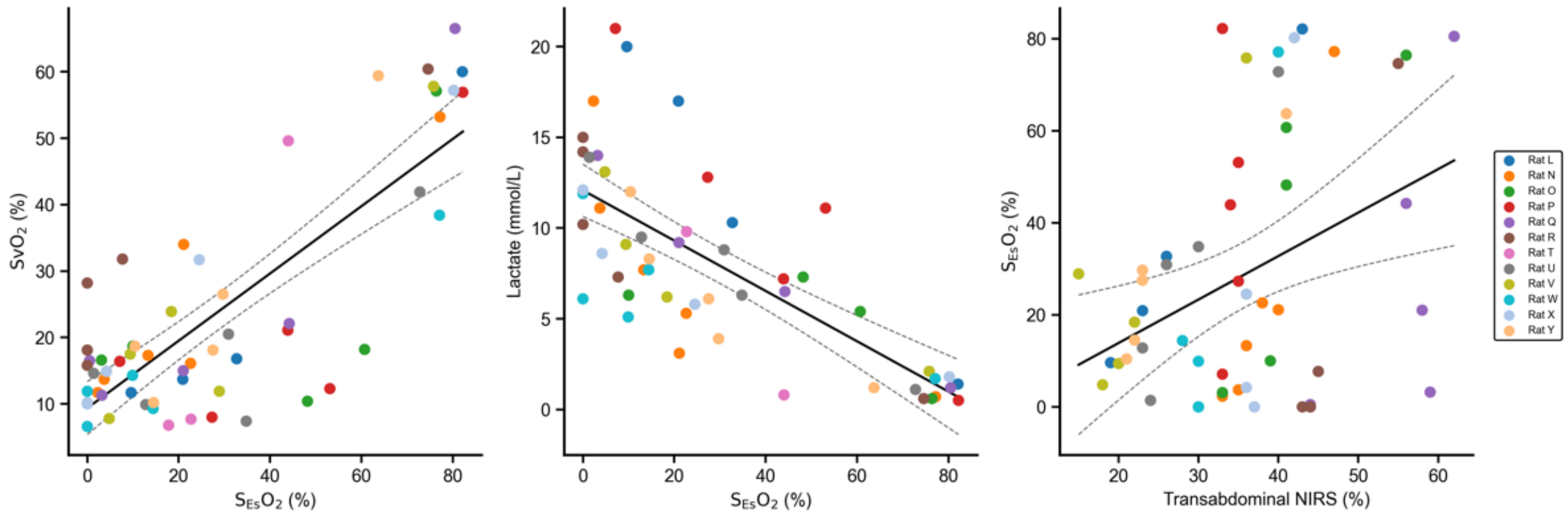

B) Hypoxia

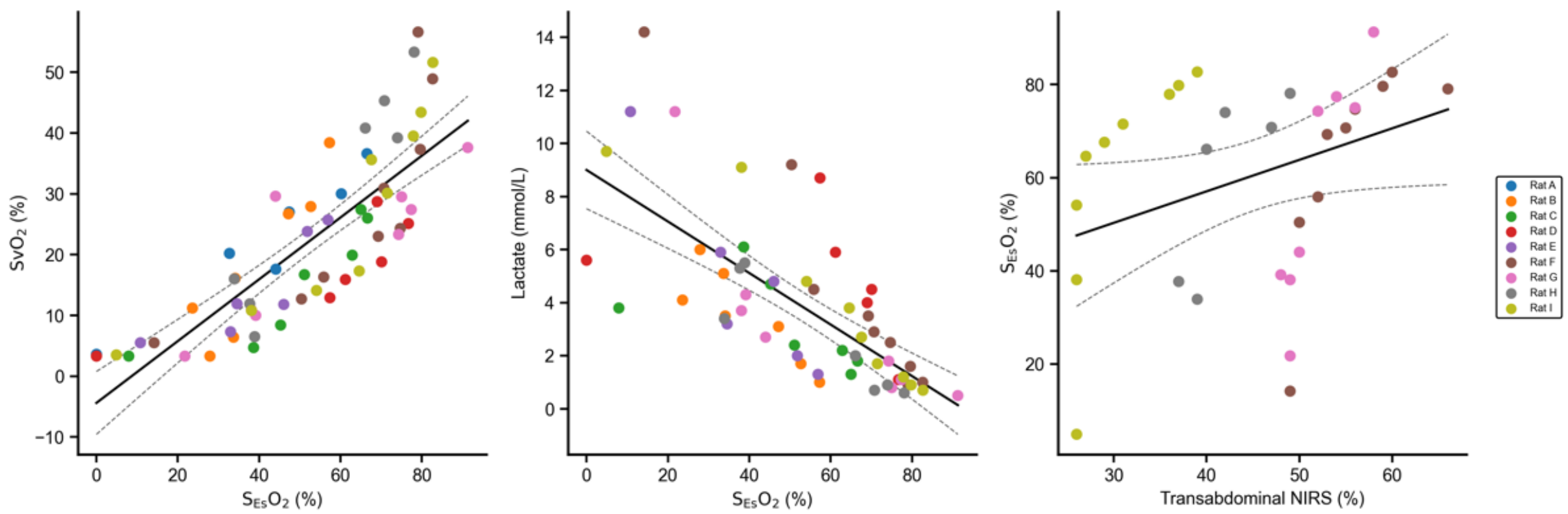

Supplemental 5.

Table 1.Diagnostic performance by Youden's test of RRS and NIRS compared to SvO_2 sampling

Model	Diagnostic	Observations (n)	Animals (n)	Optimal cut-off%	Sensitivity	Specificity	PPV	NPV
Hemorrhage	SEsO2	58	12	43.9	0.9	1.0	1.0	0.7
Hemorrhage	NIRS	56	11	39.0	0.7	0.8	0.9	0.4
Hypoxia	SEsO2	65	9	65.0	0.7	1.0	1.0	0.3
Hypoxia	NIRS	32	4	59.0	1.0	0.3	0.8	1.0
Hemorrhage (MABP≥25)	SEsO2	48	12	60.7	1.0	0.9	1.0	0.9
